# Clinical perspectives and outcomes of the giant breast phyllodes tumor and sarcoma: a real-world retrospective study

**DOI:** 10.1186/s12885-023-11279-2

**Published:** 2023-08-28

**Authors:** Naiquan Liu, Ye Kang, Ningxin Qu, Chenhui Kong, Ye Han

**Affiliations:** Department of Oncology, Shengjing Hospital of China Medical University, Shenyang, 110004 China

**Keywords:** Sarcoma, Malignant phyllodes tumor, Giant breast tumor, Phyllodes tumor, Spindle cell

## Abstract

**Background:**

Giant breast malignant phyllodes tumor or sarcoma (GBPS) are rare entities with diameter larger than 10 cm and variously histological pleomorphisms. This disease poses a significant threat to the quality of life of individuals, and its prognosis remains unclear. This study aimed to explore the differential diagnosis, treatment, and prognosis of GBPS in a real-world retrospective cohort.

**Methods:**

We collected GBPS (diameter > 10 cm, *n* = 10) and BPS (diameter ≤ 10 cm, *n* = 126) from patients diagnosed with sarcoma or malignant phyllodes tumor between 2008 and 2022. We analyzed clinical characteristics, histological status, treatment, and local recurrence using the Fisher’s exact test between GBPS (diameter > 10 cm) and BPS (diameter ≤ 10 cm) cohort. We described overall survival (OS) and disease-free survival (DFS) using Kaplan–Meier curves and identified risk factors for local recurrence using logistic regression. The tumor size, age at diagnosis, and differential immunohistochemistry markers of breast sarcoma or phyllodes tumor to determine the prognosis of GBPS.

**Results:**

In our retrospective analysis of breast malignancies, we identified 10 cases of GBPS and 126 cases of BPS, corresponding to a GBPS prevalence of 0.17% (10/6000). The median age was 38.5 years (inter-quartile range, IQR: 28.25–48.5 years). During the follow-up of period (median: 80.5 months, IQR: 36.75–122 months), the local recurrence (LR) rate was 40% and 20.6%, respectively. Clinical characteristics of young age (HR:2.799, 95%CI -00.09276—0.017, *p* < 0.05) and cytological characteristics of marked stromal atypia (HR:0.88, 95% CI 0.39–1.40, *p* < 0.05) were risk factors for the poor prognosis of GBPS by COX regression model analysis. The Kaplan–Meier curves of GBPS 5-year disease-free survival (DFS) and overall survival (OS) were 31.5 months and 40 months, respectively, and were not associated with adjuvant radiation or chemotherapy.

**Conclusion:**

We recommend mastectomy with a clear surgical margin as the preferred treatment for GBPS. Age and stromal atypia are significantly associated with recurrence. Adjuvant radiation therapy is advised; however, there was no improvement in overall survival. There is no consensus on the effectiveness of adjuvant chemotherapy and genetic methods, highlighting the need for further research into this aggressive tumor. We recommend a multidisciplinary approach involving a dedicated team for the management of GBPS.

**Supplementary Information:**

The online version contains supplementary material available at 10.1186/s12885-023-11279-2.

## Introduction

Giant breast sarcoma and malignant phyllodes tumor (GBPS) are rare diseases that may affect quality of life with rapidly progression. This type of disease was presented by case reports sparsely. Breast sarcoma (BS) is a rare and diverse group of malignant tumors that originate from mesenchymal tissue. Its incidence among breast malignancies in women is less than 1% annually, with an approximate incidence rate of 0.0046% [1, 2]. Primary BS is often associated with genetic disorders such as Li-Fraumeni syndrome, familial adenomatous polyposis, and neurofibromatosis type I [3–5], while secondary BS can occur following radiotherapy for intrathoracic cancers including non-Hodgkin lymphoma [6, 7]. Histologically, BS can be classified into various subtypes including fibrosarcoma, liposarcoma, fibromyxosarcoma, pleomorphic sarcoma, leiomyosarcoma, rhabdomyosarcoma, undifferentiated pleomorphic sarcoma, and angiosarcoma [8, 9]. Fibrosarcoma, angiosarcoma, and liposarcoma are the most prevalent subtypes of primary GBS (GBS), characterized by a rapidly increasing breast mass and a wide range of histological variations [10]. Early diagnosis and prompt management are crucial for the effective treatment of GBS. This study explores differential specialty and therapeutic dilemma in correlation to the clinicopathological characteristics in cytology and histology specimens.

Phyllodes tumor (PT) is a rare type of breast tumor that accounts for 0.3–0.9% of all breast tumors [11]. It is described by Hohannes Muller in 1838, which has lower incidence rate (1/100,000 individuals) and accounts for 0.3–0.9% of all breast tumors [12]. The histological appearance of PTs is characterized by epithelial, cyst-like voids with double-layered epithelial cells surrounded by hypercellular development in the shape of leaves [13, 14]. The majority of PTs (60–75%) are benign, with a local recurrence rate of 10–20% and a favorable prognosis [15, 16]. However, the local recurrence rate for benign PTs is 10–20%. Borderline and malignant PTs account for 15–20% and 10–20% of all PTs, respectively, with a higher local recurrence rate ranging from 15–40% and distant metastasis rate of 9–27% [12, 17, 18]. Giant PTs, which are more than 10 cm in diameter, make up around 20% of all PTs and have a higher chance of malignancy [13, 19]. The conventional treatments for PTs are wide local excision or mastectomy with sufficient clear margins. However, even with treatment, the local recurrence rate for malignant PTs is still high at 10.6%–16.1%, and the distant metastasis rate can range from 6.3–31% [20]. Therefore, further exploration of early diagnosis and effective intervention is promptly needed in reducing morbidity and mortality.

Here, we present a retrospective study of GBPS (tumor size > 10 cm) in the recent 14 years that were eventually pathologically identified as primary BS or malignant phyllodes tumor (MPT) with a median 80.5-month follow-up. The study aims to provide insights into the treatment and outcomes of GBPT in a real-world setting, as well as to compare these outcomes with those of BPS (tumor size ≤ 10 cm). This comparison can potentially help identify any differences in treatment approaches and prognosis between GBPS and BPS.

## Method

### Study design and cohort population

**Fig. 1:**
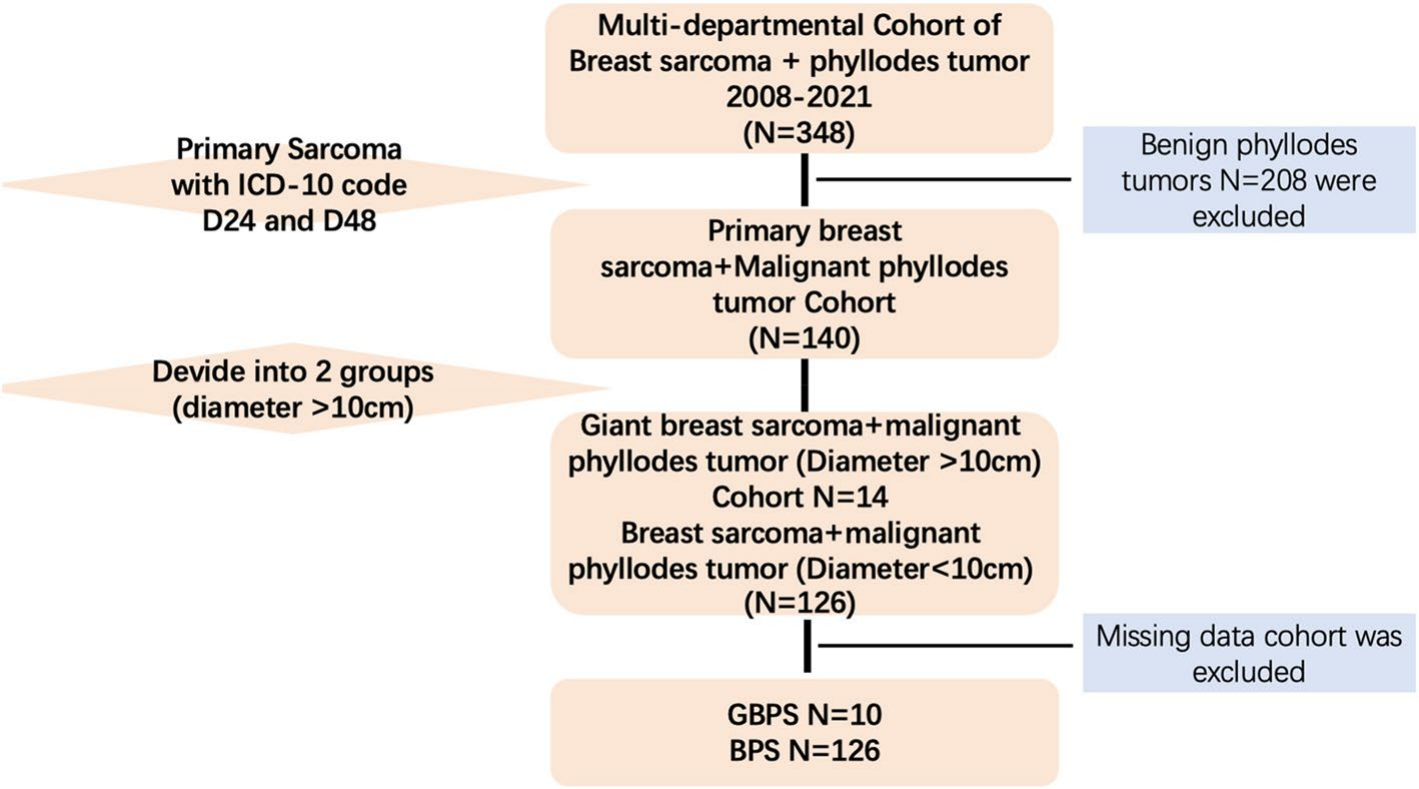
Screening flow chart

All methods were carried out in accordance with relevant guidelines. The complete NCCN Clinical Practice Guidelines in Oncology (NCCN Guidelines) for Soft Tissue Sarcoma and breast cancer provide recommendations for the diagnosis, evaluation, and treatment of STS, breast cancer as well as phyllodes tumor. We projected a retrospective cohort study including patients with pathological diagnosis of either malignant phyllodes tumor or breast sarcoma (tumor size > 10 cm) from 2008 to 2021. Patients were identified from Shengjing Hospital of China Medical University database and data was extracted from patient records. We screened the data of GBPS and BPS using the ICD-10 code D24, D48, and D48.61 from 2008 to 2021 (Fig. 1).

The criteria for the inclusion: 1 All the cases were diagnosed as either malignant phyllodes tumor or breast sarcoma from 2008 to 2021 (*N* = 348). 2 All the cases were characterized with age between 20 to 70 years old. 3 All the cases screened were divided into giant group (GBPS) and control group (BPS) in according to the diameter of tumor with a cut-off 10 cm.

The criteria for the exclusion: 1 All the cases with previous radiotherapy or radiation history were excluded. 2 The secondary tumor was excluded with any reasons in the study. 3 Benign or borderline phyllodes tumors were excluded in the study. 4 All the cases without full follow-up records were excluded (*N* = 4).

### Procedure

Patients were divided into two groups: those with a diameter larger than 10 cm (GBPS) and those with that smaller than 10 cm (BPS). Age, gender, tumor size, clinical presentation, length of symptom, history of radiotherapy, type of surgery, local recurrences, and systemic metastases were all obtained retrospectively from clinical charts and surgical data. The diagnosis of local recurrence and metastasis was confirmed by positive pathologic or imaging evidence. Follow-up data was collected by every 3–6 months patients’ visit to the hospital.

The outcome of the study was local recurrence, metastasis and death due to any cause. The survival time was defined as the time from diagnosis to disease-related death. The disease-free survival time was defined as the period between diagnosis and disease associated local recurrence.

### Statistical analysis

All statistical analyses were performed using SPSS version 28.0 (IBM Corporation, Armonk, NY, USA). Continuous variables were compared using Student’s t-test, and categorical variables were compared using the Fisher’s exact test. Five-year disease-free survival (DFS) and overall survival (OS) was analyzed by Kaplan–Meier at 5 years.

## Result

### Patients’ characteristics

Between 2008 and 2021, 6000 cases of breast tumors were recorded, among which 348 cases of phyllodes tumors and breast sarcomas were selected. We conducted a selection of 140 cases of breast sarcoma and MPT, then a division in according to the tumor size into two groups, including 10 cases of GBPS (diameter > 10 cm) and 126 cases of BPS (diameter ≤ 10 cm, missing data was excluded) with the following diagnosis: sarcoma (*n* = 23) and malignant phyllodes tumor (*n* = 113). All the patients were women, with a median age of 38.5 (IQR: 28.25–48.5) years. The distribution of 136 cases were clinically described in Table 1. The most frequent pathological diagnoses of sarcoma were fibrosarcoma (*n* = 11, 8.1%) and liposarcoma (*n* = 12, 8.8%). The local recurrence rate of were 30% (*n* = 3) and 20.6% (*n* = 26) in GBPS and BPS, respectively. Table 2 provides the pathological characteristics by GBPS and BPS. No significant bias was identified between GBPS and BPS. The clinical characteristics, including age at diagnosis, tumor size, axillary treatments, nuclear grade, adjuvant therapy were balanced in the two groups. However, the surgical managements varied significantly between GBPS and BPS group, and mastectomy accounted for 100% and 27.8% respectively (Table 2). Background cellularity and background cell atypia were seen in most cases (60% and 80%), which is closely associated with local recurrence.

**Table 1:** Clinical characteristics of the cases

Patients characteristics (*n* = 10 vs. 126)			
	GBPS	BPS	*p*
**Age (years)**			
20–39	5	32	
40–59	4	81	0.07
60–69	1	13	
**Development (months)**			
< 3	3	2	0.61
> 3	7	12	
**Location of tumor**			
Left	5	66	0.07
Right	5	60	
**Skin Invasion**			
Yes	1	0	0.52
No	9	126	
**Diagnosis**			
Malignant Phyllodes Tumor	6	107	0.06
Liposarcoma	2	10	
Fibrosarcoma	2	9	
**Operation**			
Wide local excesion	0	57	0.098
Mastectomy	10	35	
**Axillary treatments**			
No	2	111	0.07
SLNB	3	11	
ALND	5	4	
**Adjuvant therapy**			
Chemotherapy	1	1	0.89
Radiotherapy	4	1	
**Remote metastasis**			
Lungs	1	0	
kidney	1	0	
**Local recurrence**			
Yes	3	26	0.77
No	7	100

**Table 2:** Pathological features of the cases

Cytological features (*n* = 10 vs. 126)			
	GBPS	BPS	*p*
**Pathological diagnosis**			
Fibrosarcoma	2	16	0.07
liposarcoma deriving from phyllodes	2	10	
Malignant phyllodes tumor	6	100	
**Shape of background cell nuclei**			
Plump	6	4	0.20
Spindle	3	8	
Lobular	1	2	
**Background cellularity**			
marked	6	78	0.07
moderate	2	39	
mild (pleomorphism)	2	9	
**Stromal Overgrowth**			
Low	5	52	0.83
High	5	74	
**Proportion of spindle cells**			
> 30%	2	6	0.55
10–30%	2	2	
**Background adipocytic morphology**			
Regular	6	13	0.12
Pleomorphism	4	1	
**Background cell atypia**			
Low	2	58	0.52
High	8	68	
**Background cell mitosis**			
Low (< 5/10HPF)	0	88	0.73
High (> 5/10HPF)	4	38	
**Nucleur necrosis**			
Low	5	114	0.25
High	5	12	
**Bleeding with infiltration**			
Yes	1	15	0.174
No	9	111	

### Survival analysis and cox proportional model

**Fig. 2:**
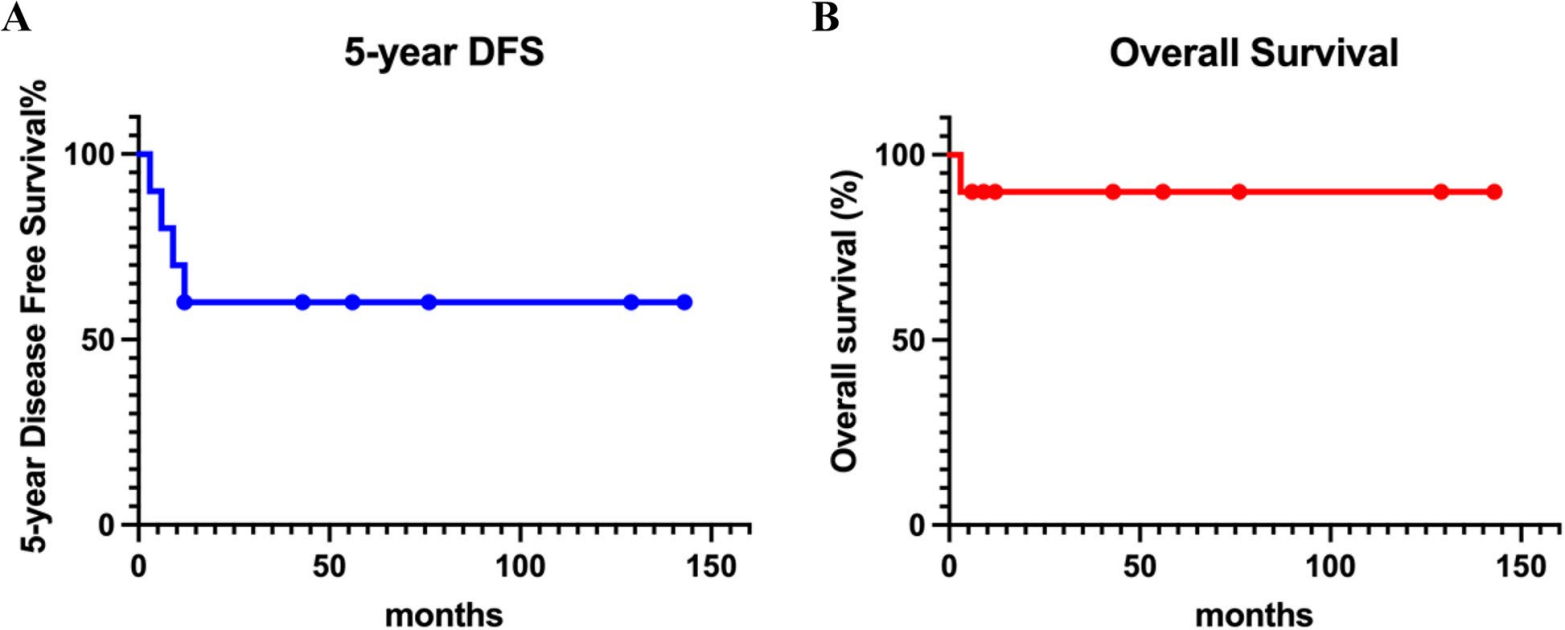
Kaplan–Meier survival curves for of the GBPS. **A** Disease free survival (DFS) is analyzed in Kaplan–Meier curves for a 5-year follow-up. **B** Overall survival (OS) is analyzed in Kaplan–Meier curves for a 5-year follow-up

**Fig. 3:**
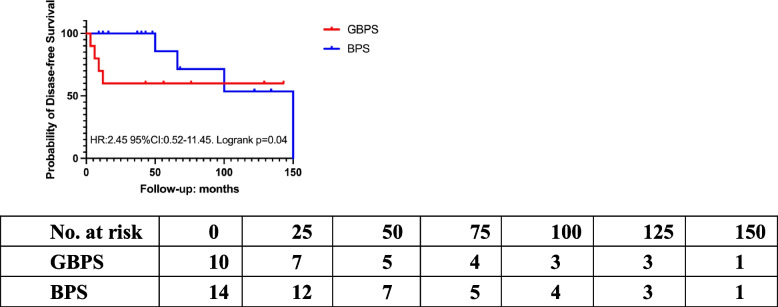
Kaplan–Meier survival curves of DFS between GBPS and BPS. There is significantly survival difference between GBPS and BPS (*p* < 0.05)

**Fig. 4:**
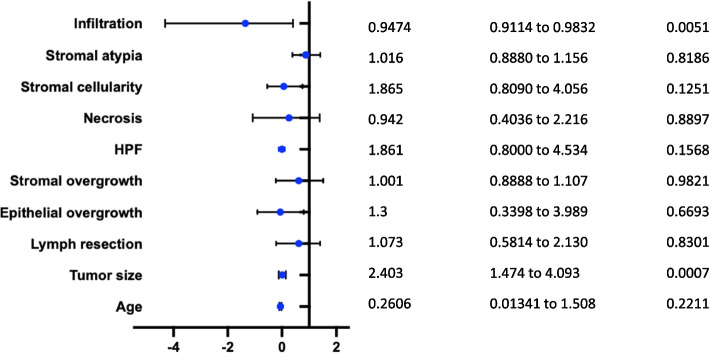
Forest plot of the association between risk factors and local recurrence rate of GBPS. Hazard ratios for local recurrence with 95% confidence interval (CI) and *p*-values analyzed by a Cox proportional model. Squares represent study-specific relative risk, horizontal lines indicate the 95% CI

The survival rate of 5-year DFS and OS of GBPS were analyzed as 60% and 90% by Kaplan–Meier analysis (Fig. 2). In the cohort of GBPS, the 5-year survival rate was no longer than that of BPS (HR:2.45, 95%CI 0.05–11.45, *p* < 0.05) by Kaplan–Meier analysis (Fig. 3). We further carried out risk predictors analysis and interaction between characteristics and prognosis. By Cox regression analysis, the risk factors for local recurrence or metastasis were significantly associated with age (HR:2.799, 95%CI -00.09276—0.017, *p* < 0.05) and stromal atypia (HR:0.88, 95% CI 0.39–1.40, *p* < 0.05) (Fig. 4).

### Typical GBPS cases

**Fig. 5:**
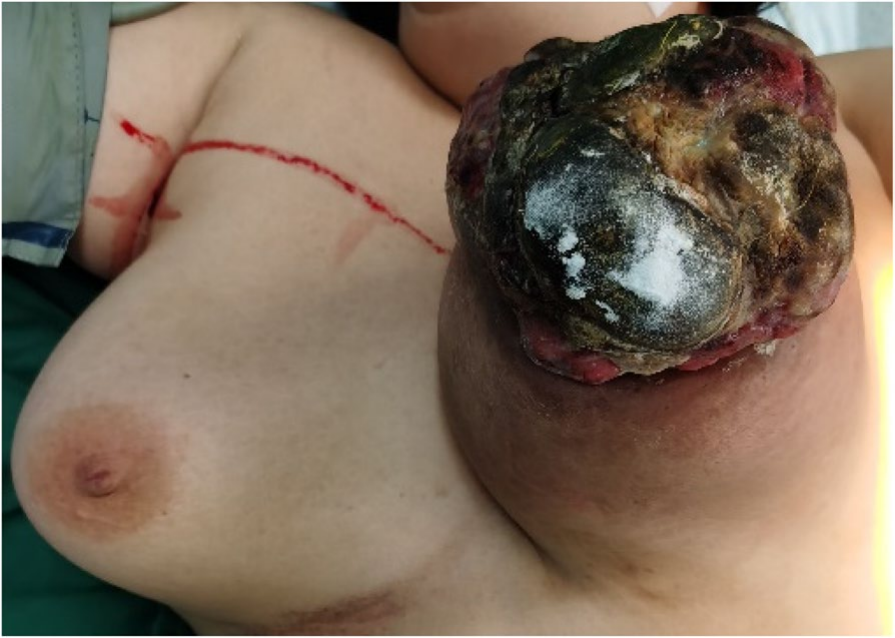
This image depicts a giant tumor of the left breast with bleeding and ulcers from the case with the worst prognosis. The gross picture of giant tumor with internal necrosis, 23 × 19 × 13 cm in size

**Fig. 6:**
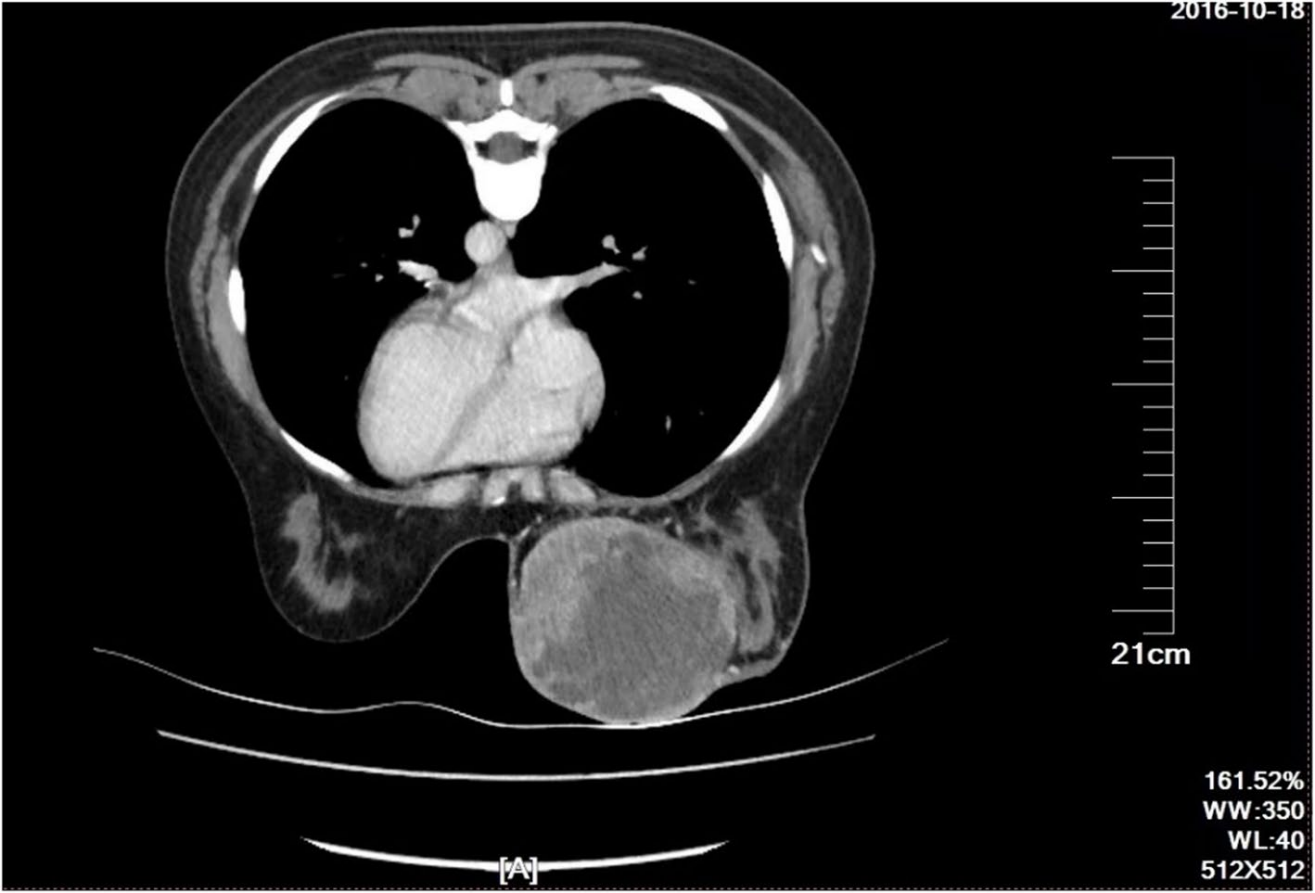
CT scan of the breast tumor. Breast CT revealed an irregular mass (12.0 × 11.3 × 11.7 cm) with local mixed echoes and marginal vessels formation. Left axillary lymph nodes are enlarged to 2.5 × 1.2 × 0.8 cm

**Fig. 7:**
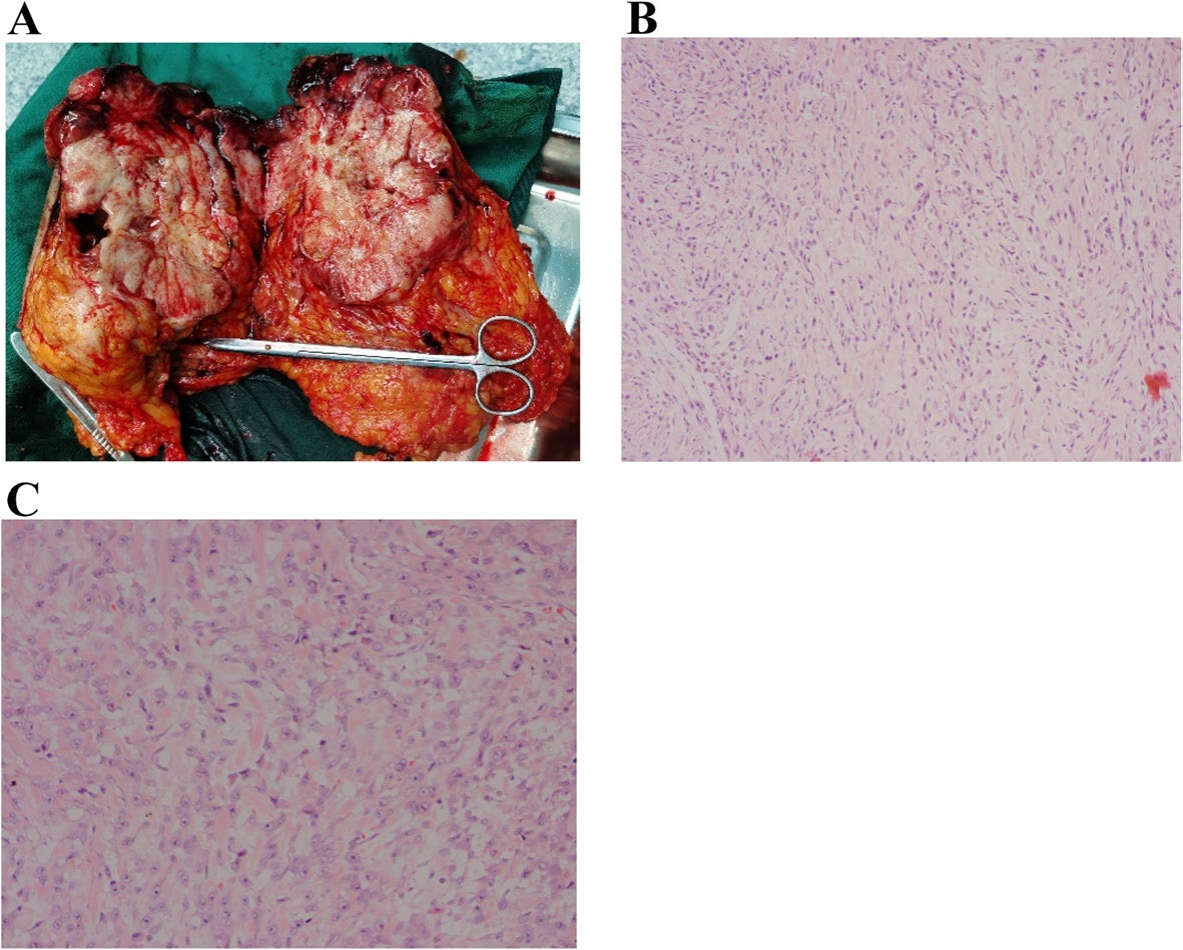
Gross picture and microphotograph of the giant tumor. **A** Gross image of the giant tumor with internal necrosis, 23 × 19 × 13 cm in size. **B** Histopathology of a core needle biopsy of the tumor revealed multiple fusiform atypical tumor cells (Hematoxylin and eosin, original magnification, 100 ×). ER (-), PR (-). **C** A pathological microphotograph revealed irregular fusiform tumor cells with a variable nucleus and numerous microcapillaries. (Hematoxylin and eosin, original magnification, 200 ×). Mitotic 8/10HPF

**Fig. 8:**
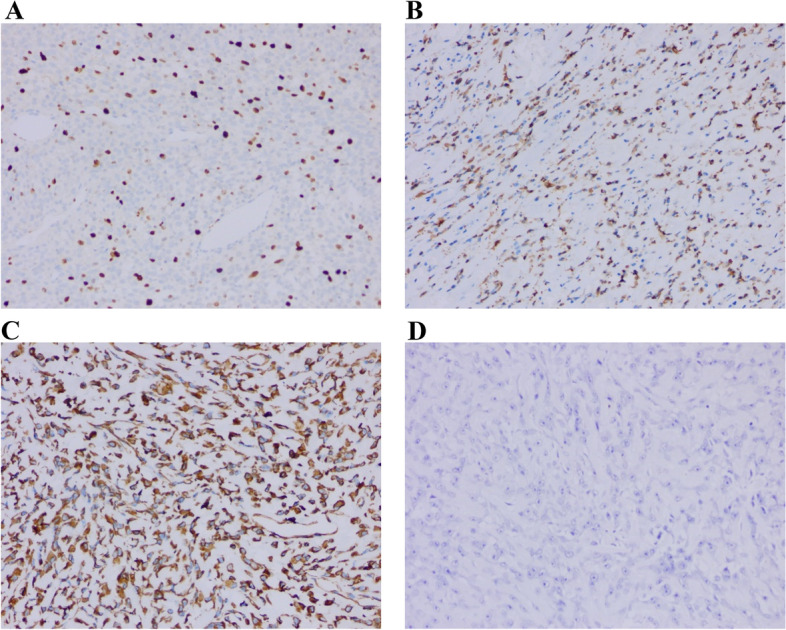
Hematoxylin and eosin staining of the giant sarcoma (original magnification, 200 ×). **A** Immunohistochemistry staining revealed positive of Ki67. **B** SMA with weak positive staining. **C** Vimentin with positive staining. **D** Bcl-2 with negative result

The typical giant breast sarcoma with poor prognosis was described in the cohort, left breast of the patient beard a 24 × 23 cm firm lobulated and ulcerated mass (Fig. 5), torn skin with dripping bleeding, which had a unilateral, fast-growing process (average 3.25 months) with palpable mass in the clinic (Fig. 5). The case belongs to GBPS with poor results of renal failure three months after surgery and death. In this study, the tumor's location is balanced in two groups. A breast sarcoma CT scan image revealed a 17 × 14.7 × 14.3 cm solid mass with irregular echoes and calcifications on the left breast (Fig. 6). A core needle biopsy pathologically revealed that the tumor was a low-grade sarcoma arising from phyllodes tumor cells (Fig. 7A, B, C). All the cases undergone mastectomy with axillary dissection or sentinel lymph nodes biopsy in the treatment of GBPS. The following are the immunohistochemical staining results (Fig. 8 and Supplement 1): Vimentin ( +), SMA (weak +), S-100(-), CK (-), P63 (-), Bcl-2(-), Stat6(-), MelanA(-), EMA (-), β-catenin (-), Ki67(40–90% +).

In our records, only one case of adjuvant chemotherapy was administered, and two patients completed radiotherapy after the operation in GBPS group. The follow-up was recorded on a regular basis every 3 months. One serious case was unable to get more data due to a rapid relapse with renal failure and finally died in 3 months after operation. Within 3 years of the initial diagnosis, there was one case of distant metastasis.

## Discussion

In this study, we evaluated the clinical characteristics and histopathological features of giant breast sarcoma and malignant phyllodes tumor (GBPS) in one institution. To our knowledge, GBPS are rare entities and impair the quality of life seriously. There are only no more than 20 cases reports of giant breast sarcoma or malignant phyllodes tumors in the PubMed when sorting with ‘giant breast tumor’, ‘giant breast sarcoma’ and ‘giant phyllodes tumor’. There is limited information available on the diagnosis, prognosis, and treatment of GBPS because of rarity with only 1% of all cancers [5]. Our cohort from a real-world study proved a mastectomy is meaningful, but the LR rate is still as high as 30%. The 5-year DFS and OS in our study is relatively 60% and 90% by Kaplan–Meier method. It is meaningful to explore the proper treatment and risk factors of GBPS.

The incidence of GBPS in women without metastases was found to be around 0.07% (4/6000) in this investigation. Previous study reported that duration for the GBPS to grow ranges from 2 to 10 months, with an average of 4.75 months, partially with pain and bleeding [21]. To obtain a wide and clear margin, total mastectomy has traditionally been considered the gold standard surgical therapy for breast sarcoma, especially for tumors larger than 10 cm. In our cohort all the GBPS undergone mastectomy instead of operation with wide margins, that is significantly different with BPS(*p* < 0.05). Of note, 50% of the participants had enlarged lymph nodes at the time of preoperative examination, no lymphatic metastases were found in the final pathology report. Even though the management of the axilla is still unclear, some subtypes of sarcoma are suspect of having the capacity to disseminate lymphogenously [75]. There are reports of lymphogenous spread in angiosarcoma, rhabdomyosarcoma, and epithelioid sarcoma^1^. Many previous researches perform axillary lymph node biopsy or dissection for breast sarcoma or malignant phyllodes tumors [22]. Lee JS, et al. reported 25% of BS had lymphatic metastases if imaging suggested lymphadenopathy [23]. As a preventative measure against undiscovered early lymph node metastases, we therefore performed axillary lymph nodes dissection (ALND) or sentinel lymph node biopsy (SLNB) in according to the enlarged lymph nodes revealed by imaging, that pathologically diagnosed no metastasis. Malignant PTs (MPT) have a metastasis rate of about 22% and can spread to the pancreas, lung, kidney, and duodenum [18, 24]. With only three examples of PTs spreading to the stomach and causing anemia, MPT metastasizing to the gastrointestinal tract is rare [25–27]. The most severe case in our cohort, which had a similar prognosis to those previously reported, passed away 3 months after surgery from a large tumor with 17 cm diameter and skin ulceration. In this case, the abdomen and lung were examined using a contrast-enhanced CT scan; no metastases were discovered. However, due of the tumor's enormous size and quick metastasis, we did not have the chance to give a molecular or genomic test to predict tumor behavior.

The GBPS is uncommon cancer subtype with distinct heterogeneity. The distinction between GBS and MPT is made by residual epithelial tissue and malignant mesenchymal components, but with a similar treatment and prognosis. In a retrospective analysis that ranged from the years 1991 to 2014, BS can be divided as undifferentiated pleomorphic sarcoma (70.6%), angiosarcoma (17.6%), osteosarcoma (5.9%), chondrosarcoma (5.9%), fibrosarcoma (0.1%), and liposarcoma (0.1%) [28]. In a separate investigation of 991 BS cases, spindle cell, leiomyosarcoma, and giant cell sarcomas were all reported to be 13.4%, 11.7%, and 10.1%, respectively [7]. GBS has tumor-specific differentiation in the stromal nuclear pleomorphism but neither squamous differentiation nor epithelial cells [76]. Histologically, undifferentiated pleomorphic sarcoma has a storiform, fascicular structure with a high level of cellular atypia and pleomorphism, whereas liposarcoma has variable adipocytic differentiation and heterogeneous shape embedded in a vascularized stroma. In addition, decisive immunohistochemistry markers for undifferentiated pleomorphic sarcoma include S100/SOX10, smooth muscle actin (SMA), and desmin [29, 30]. All GBPS in this study—two fibrosarcomas and two liposarcomas—showed adipocytic pleomorphism and a significant amount of cellular atypia with mitosis. Two cases of fibrosarcoma composed of spindle cell and bleeding with inflammatory infiltration which manifested poor prognosis. The majority of GBPS includes the specific hallmarks, including mitoses, pleomorphism, necrosis, and inflammatory infiltration, as this study demonstrates (Table 2). Previous studies demonstrated characteristics in GBPS as improved stromal cellularity, stromal nuclear pleomorphism, stromal overgrowth, more than 10 mitoses per 10 HPF, and an infiltrative boundary [18].

We used a variety of immunohistochemical stains to corroborate the final diagnosis, however only three gave strong or positive results, as indicated below (Supplement 1): S-100 (-), Bcl-2 (-), Stat6 (-), MelanA (-), EMA (-), Calponin (-), CD10 (-), CD34 (-), CK (-), Desmin (-), HMB-45 (-), P63 (-), and β-catenin (-) were all negative. However, Ki67 (40–90% +), SMA (weak +), and Vimentin ( +) were positive in GBS. Due to the markers SMA (weak +), CK (-), and Desmin, the relationship between the metabolic mechanism of the islet cell and giant sarcoma is not as strong (-). Immunohistochemistry findings of diffuse cytokeratin (-) and p63 (-), which support a diagnosis of non-metaplastic carcinoma, must be separated from metaplastic carcinoma to identify BS. The relative risk of mortality is 5.12 (*p* = 0.0022), which is unsatisfactory and indicates that 32.2% of patients have a poor prognosis [31]. There may be focal expression of CKs and P63 as supporting markers in MPT, but none do in this example [32]. Vimentin, a classic marker for aggressive and invasive mesenchymal cells, most likely predicted the rapid development and early relapse of the giant sarcoma [33]. For GBPS, a previous study investigated the large tumor size with skin ulceration had significant poor prognosis [77], which is proved in our cohort. GBPS has a poor prognosis due to high mitoses (ten or more per 10 HPF), infiltrative borders, stromal atypia, marked stromal cellularity, and overwhelmed stromal development [34–36].

Although they remain unidentified, the genetic variations of GBPS are being carefully examined. In PT stromal cells, there is a unique CD34 activity that is associated with benign histology and is recognized as being negative in MPT differentiation [37]. A few indicators, including bcl-2 and CD117, have been identified as sarcoma-specific chromosomal abnormalities and have increased molecular adjunction in MPT [37–40].

With the expression of pluripotency factors such as OCT3/4, NANOG, KLF4 and SOX2 [41–43], stemness in sarcoma is a variable condition. By investigating genetic alterations in p53, Ki67, CD117, EGFR, p16, and VEGF, numerous research have sought to improve MPT categorization and prognosis; however, only p53 expression and the Ki67 index have been proven to be significantly linked with DFS and OS [44–46]. Recently, it was found that MED12 is commonly changed in the initial phases of PT [47]. Notably, genetic mutations in FLNA (28%), SETD2 (21%), and KMT2 (9%) were exclusively found in PT, indicating that it is a distinct component from BS. The aggressive biologic behavior in PT was discovered to be caused by mutations in p53, RB1, and NF1, as well as genetic amplification of EGFR and IGF1R, suggesting EGFR could be a therapeutic target for MPT. P53, on the other hand, has been identified as mutated in 15% of sarcomas, which can be targeted or regulated in an early stage of clinical trial by blocking the interaction of p53-MDM. In 4.2% of pleomorphic liposarcomas and about 10% of leiomyosarcomas, RB1 mutations have been reported. Isocitrate dehydrogenase (IDH) is a particular enzyme that takes part in the oxidation of NAPDH throughout the metabolic process in sarcomas. IDH was found to have a mutation in early research, supporting its status as a prospective target for the differential diagnosis and therapy of BS, but no antibody approvement in clinic. The literature highlights the possibility of targeting the extracellular region of EGFR as a potential approach for anti-EGFR drug discovery. Currently, monoclonal antibodies like cetuximab and panitumumab are used to target EGFR in certain cancer types, but there is no specific mention of these antibodies being approved for GBPS [48]. Curcuma has been used as an adjuvant treatment for osteosarcoma as a target of EGFR, but not available for breast sarcoma. Clinical trials and research studies are continuously exploring potential treatment options to improve patient outcome for GBPS.

The prognosis of GBPS is influenced by its clinicopathological characteristics, including tumor size greater than 5 cm, high-grade nucleus, stromal atypical cells, and positive resection margins [49]. According to official statistics, the median 5-year overall survival rate for sarcoma is 63.5% [50], which is similar to our results of GBPS (5-year DFS 60%). In contrast to other forms of breast malignancies, breast sarcomas are often staged and treated differently. In a meta-analysis of 10 retrospective studies that included 9 reports of local recurrence rates, 4 reports of distant metastasis, and 4 reports of survival, Thind A. et al. found that tumor size (*p* = 0.03) and a surgical margin of at least 1 cm did not significantly affect local control (*p* = 0.33, *n* = 456), distant metastasis (RR 1.93, 95% CI 0.35—10.63; *p* = 0.45, *n* = 72), or overall survival (RR 1.93, 95% CI 0.42—8.77; *p* = 0.40, *n* = 58) [51]. The local recurrence and metastatic rates of MPT range from 15 to 40% and 9% to 27%, respectively. Once metastasis occurs, the prognosis is dismal, with death occurring within the next two years [14, 52, 53]. According to the current study's findings, GBPS has a recurrence rate of 30% and an early metastasis rate of 20%, which is higher than that of MPT or BS. In our cohort we concluded a similar result as LR of 30%(*n* = 3) of GBPS, as well as that of 20.6%(*n* = 26) of BPS.

Regarding adjuvant therapy for GBPS, there is a lack of consensus. Adjuvant radiation for MPT remains controversial (category 2B), according to the National Comprehensive Cancer Network (NCCN) guidelines for the treatment of PTs (version 4, 2022) [54]. However, the use of radiotherapy has lately increased due to the substantial risk of recurrence in several reported cases [55]. A retrospective study of 59 individuals with soft tissue sarcoma over a 43-year follow-up found that 4 of 16 patients received radiation after segmental resection and 13 of 38 patients received radiation after mastectomy. Local recurrence occurred in 13% of patients who underwent mastectomy and radiation, compared to 13 individuals (34%) who underwent mastectomy alone. Without adjuvant radiation, local failure occurred in 60% of instances with positive surgical margins, while local recurrences affected more than 75% of patients who underwent surgery alone [56]. Radiotherapy was successful in treating high-grade GBS according to Johnstone et al., who studied mastectomy and postoperative radiotherapy in ten patients over the course of 99 months [9]. Adjuvant radiation therapy is recommended for borderline and malignant PTs with a tumor-free margin of 1 cm or greater [57]. Adjuvant radiation is recommended after R1 resection and indicated after R0 resection for high-risk malignant tumors (higher grade, size > 5 cm) [58]. Radiation-induced secondary sarcoma limits the clinical application of radiotherapy. Palta et al. advised hyper fractionated and accelerated radiotherapy (HART) for radiation-induced breast angiosarcomas, and they found that 14 patients who received HART had a 64% 5-year disease-free survival rate [59]. In a median follow-up of 76 months (range: 7–216 months), postoperative radiation had no impact on cancer-specific survival, regardless of mastectomy or breast conserving surgery [60, 61]. According to a multivariate Kaplan–Meier curves and Cox proportional hazards analysis of 1353 MPT patients with a follow-up of 99 months (range: 0–331 months), adjuvant radiation does not increase overall survival [52]. There were limited studies specifically addressing the effect of site and radiation dose (in Gray, GY) on the OS and DFS of patients with malignant phyllodes tumors receiving adjuvant radiotherapy. The impact of radiation dose and site (field) on OS and DFS can vary depending on several factors, including the extent of tumor spread, patient characteristics, and the overall treatment plan. Previous studies reported the inclusion of all relevant lymphatic drainage sites in the radiation volumes seemed to result in the best therapeutic effect [62]. It is essential to consider that higher radiation doses may increase the likelihood of achieving local tumor control but also carry the potential risk of radiation-related side effects. The radiation field (site) will depend on the extent of the original tumor and the surgical procedure performed. McGowan et al. [2] reported that the cause-specific survival, which received over 48 Gy radiation dose comparing with the group of no or less than 48 Gy radiation dose, was 91% and 50%, respectively. However, to determine the specific impact of site and radiation dose on OS and DFS in malignant phyllodes tumor patients, larger clinical studies with sufficient follow-up and comprehensive patient data are required.

Adjuvant radiotherapy is still under consideration for its fear of the potential late effects of cumulatively high radiation dose, including rib fractures, lung fibrosis and cardiomyopathy. In addition, radiotherapy is limited in treatment of GBPS due of the rarity of radiation-induced breast sarcomas (RIBS), which accounts for less than 1% of all initial breast malignancies. Angiosarcoma is the most frequent subtype of RIBS, which makes up around one-third of all BS cases with presented at 5^th^ or 6^th^ decade of life [63]. Radiation-induced breast angiosarcoma (RIBA) exhibited an incidence of 0.1% after breast-conserving therapy, which derives from the dermis of irradiated breast. Surgery with negative margins is the main treatment for localized disease [64]. According to earlier investigations, RIBS had a mastectomy as a strong recommendation in most series. HART, or hyper-fractionated accelerated RT, may be very helpful for RIBS [65]. However, tumor margins are technically difficult to be assessed for GBPS to ensure clear margins, due to infiltrative margins and multifocality. Adjuvant radiotherapy is proposed by some groups for GBPS [66].

Chemotherapy for BPS is reported infrequently and with uncertainty. In this study, chemotherapy was advised for two cases. Some studies on adjuvant chemotherapy in BS yielded inconclusive results [7, 67, 68]. Zer A, et al. performed a comprehensive review and meta-analysis of 5044 cases reported between 1974 and April 2016 to evaluate the advantages and disadvantages of multi-agent chemotherapy in advanced soft tissue sarcoma. Multi-agent chemotherapy was shown to be linked with a marginally significant increase in overall survival (HR:0.79, *p* = 0.02) and a marginally significant increase in progression-free survival (PFS) (HR:0.86, *p* = 0.05) [69]. Adjuvant treatment was shown by Gutman et al. to be associated with longer DFS in patients from the MD Anderson Cancer Center with breast sarcoma. An ifosfamide + doxorubicin regimen gave a minor improvement in soft tissue sarcomas with respect to OS, LR, and metastasis, according to a comprehensive assessment that included 22 trials and 1953 participants. A Japanese patient was given neoadjuvant chemotherapy and a mastectomy for a 10 cm leiomyosarcoma with a 12-month follow-up. According to the findings of the study, neoadjuvant chemotherapy for aggressive high-grade lesions is a viable therapeutic approach with promising outcomes [70]. Neoadjuvant chemotherapy's future, however, is still up in the air due to the rarity and lack of clarity surrounding the measurement of sarcoma regression.

The genetic target therapy is recommended in some series with GBPS. Receptor tyrosine kinases (RTK), including as PDGFR, have been demonstrated to be expressed or elevated at the mRNA level in certain BS in addition to VEGF/VEGFR. Pazopanib, a tiny multi-targeted tyrosine kinase inhibitor (TKI) against VEGFR1, VEGFR2, VEGFR3, and PDGFR, has been given the green light for the treatment of metastatic BS following anthracycline-based chemotherapy or as a first line of therapy for patients who are ineligible for this treatment [78]. Pazopanib, a tiny multi-targeted tyrosine kinase inhibitor (TKI) against VEGFR1, VEGFR2, VEGFR3, and PDGFR, has been given the green light for the treatment of metastatic BS following anthracycline-based chemotherapy or as a first line of therapy for patients who are ineligible. Another multikinase inhibitor, sorafenib, targets the kinases Raf, PDGF, VEGFR2, VEGFR3, and c-Kit, was only proven to be effective against angiosarcoma in a phase 2 trial, with 5/37 patients exhibiting at least a partial response. Though no RECIST responses were identified in the phase II S050 study of sorafenib in selected sarcoma histotypes, including five angiosarcomas, the majority of angiosarcoma showed a clinical benefit. A limited investigation of RIBS, including RIBA, showed a positive marker for Kit RTK by IHC that is not coupled with exon 11 mutations, that provides a rationale for imatinib usage in RIBS. A novel weapon is immunotherapy (IO) conjugated with chemotherapy as a systematic regimen. Compared to other cancer types, primary breast angiosarcomas had a higher mutational rate, along with a 45% positive for PD-1/PD-L1 and tumor-infiltrating lymphocytes [71]. There are totally 20 clinical trials of PD-1/L1 inhibitors plus chemotherapy registered in clinicaltrials.gov in the treatment of sarcomas. The effective prediction of IO, including high PD-L1 expression, microsatellite instable-high/mismatch repair deficient phenotype, tumor mutation burden-high status, are proposed without solid evidence in evaluating prognosis. Obviously, the combination therapy is a promising treatment for BS, which needs further exploration.

In this study, the median 5-year DFS and OS of GBPS are 60% and 90%, respectively. A recent study found that a quickly progressing tumor with significant skin invasion and ulceration ultimately led to tumor lysis and death [72]. There was no statistically significant difference in survival rates between GBS and MPT according to several studies. In comparison to the 5-year OS, which was 86.5 and 78.5%, the 5-year DFS for BS and MPT was 59.1% and 57.4%, respectively (*p* = 0.816) [28]. Both BS and MPT diagnosed at department of pathology in Singapore General Hospital from January 1991 to December 2014 have a poor prognosis in the median follow-up of 37.86 months (range: 0.13–272.82 months) when it comes to multifocality (*p* = 0.019), histological subtype (*p* = 0.014), the presence of malignant heterologous elements (*p* = 0.001), and surgical margin (*p* = 0.023) [28]. The 5-year retrospective investigation of Asian patients' Kaplan–Meier curves revealed no appreciable difference in 5-year DFS and OS in 35 cases of BS and 70 cases of MPT [73]. According to published studies, BS has a poorer prognosis than breast cancer. The 5-year DFS and OS were 44% and 55%, respectively, according to a retrospective study of 103 breast sarcomas with a median size of 4.45 cm (range: 0.8–22 cm) [68]. The prognosis is not affected by pathological differentiation of GBS and MPT, in this case, our GBPS and BPS cohorts are reasonable. The 5-year OS was verified to be 67% (83% for tumor size 5 cm and 42% for tumor size > 5 cm) in another retrospective analysis involving 13 instances of primary BS with mastectomy. Tumor size (> 5 cm), residual tumor and margin, cellular pleomorphism, and angiosarcoma were proved to be prognostic factors for survival rate [23, 74]. Age and histopathological stromal atypia are independent predictors of local recurrence rate in our cohort by Cox proportional analysis. In addition, mitoses and giant sarcoma with the broken skin barrier are associated with exceptionally high death and recurrence rates in this study. This study fills in the knowledge gaps in our understanding of GBPS and encourages further investigation of the underlying principles.

### Limitation

The retrospective study conducted has certain limitations. It involved a systematic investigation of the rare Giant Breast Sarcoma (GBPS) by comparing 136 cases of breast malignancies (GBPS vs. BPS: 10 vs. 126) from a single institution, potentially introducing selection bias. While two co-authors collected the data together to minimize bias, the sample's representativeness remains questionable due to its sole source from one institution. The small sample size and rarity of GBPS pose challenges in drawing definitive conclusions. However, in an effort to understand its characteristics better, we divided the cases into the giant group and normal group, ensuring a balanced representation. Despite this approach, the limited comprehension and uncertain prognosis of GBPS call for further investigations to shed light on this uncommon and diverse condition.

Our research contributes to filling the existing knowledge gap surrounding GBPS and emphasizes the need for greater attention and consolidation of efforts to study this rare and complex medical condition.

## Conclusion

The GBPS are extremely rare and highly invasive, that requires surgical mastectomy as the critical treatment without lymph nodes resection. The GBPS with features, including age, stromal atypia and mitoses are associated with further metastasis and poor prognosis. Adjuvant radiotherapy and chemotherapy for high-grade BS cancer are both controversial. For a better clinical outcome, each case of GBPS should be collaborated by a multidisciplinary team in a dedicated facility. A multidisciplinary discussion in a dedicated center and a contrast-enhanced CT are necessary for an accurate evaluation and diagnosis.

Given the rarity of GBPS, collaborative efforts among multiple institutions or international registries may be necessary to accumulate enough data for meaningful analysis. Advancements in molecular profiling and understanding of the genetic and molecular characteristics of these tumors may lead to the development of targeted therapies that can specifically address the unique features of GBPS. In addition, immunotherapeutic approaches, such as immune checkpoint inhibitors and adoptive T-cell therapy, have shown promise in treating various types of cancer. Moreover, combinations of different treatment modalities, such as surgery, radiation therapy, chemotherapy, targeted therapies, and immunotherapies, may be explored to improve treatment outcomes and overall survival rates. The field of oncology is continuously evolving, and new breakthroughs may emerge that could significantly impact the treatment landscape for GBPS.

### Supplementary Information

**Additional file 1: Supplement 1.** Hematoxylin and eosin staining of the giant sarcoma with negative result (original magnification, 200×)

**Additional file 2.**

## Data Availability

All the data included in the manuscript can be found and shared publicly. All the methods were carried out in accordance with relevant guidelines and regulations. The datasets used and/or analysed during the current study available from the corresponding author on reasonable request.

## References

[1] Abdou Y, Elkhanany A, Attwood K, Ji W, Takabe K, Opyrchal M (2019). Primary and secondary breast angiosarcoma: single center report and a meta-analysis. Breast Cancer Res Treat.

[2] McGowan TS, Cummings BJ, O’Sullivan B, Catton CN, Miller N, Panzarella T (2000). An analysis of 78 breast sarcoma patients without distant metastases at presentation. Int J Radiat Oncol Biol Phys.

[3] Pfister DG, Rubin DM, Elkin EB (2015). Risk adjusting survival outcomes in hospitals that treat patients with cancer without information on cancer stage. JAMA Oncol.

[4] Malkin D, Li FP, Strong LC (1990). Germ line p53 mutations in a familial syndrome of breast cancer, sarcomas, and other neoplasms. Science (New York, NY).

[5] Lahat G, Lazar A, Lev D (2008). Sarcoma epidemiology and etiology: potential environmental and genetic factors. Surg Clin North Am.

[6] Fernebro J, Bladström A, Rydholm A (2006). Increased risk of malignancies in a population-based study of 818 soft-tissue sarcoma patients. Br J Cancer.

[7] Lee JS, Yoon K, Onyshchenko M. Sarcoma of the breast: clinical characteristics and outcomes of 991 patients from the national cancer database. Sarcoma. 2021;2021. 10.1155/2021/8828158. 10.1155/2021/8828158 PMC7843167 33542674

[8] Duncan MA, Lautner MA (2018). Sarcomas of the Breast. Surg Clin North Am.

[9] Johnstone PA, Pierce LJ, Merino MJ, Yang JC, Epstein AH, DeLaney TF (1993). Primary soft tissue sarcomas of the breast: local-regional control with post-operative radiotherapy. Int J Radiat Oncol Biol Phys.

[10] Li GZ, Raut CP, Hunt KK, Feng M, Chugh R (2021). Breast sarcomas, Phyllodes tumors, and Desmoid tumors: epidemiology, diagnosis, staging, and histology-specific management considerations. Am Soc Clin Oncol Educ Book Am Soc Clin Oncol Ann Meeting.

[11] Pitsinis V, Moussa O, Hogg F, McCaskill J (2017). Reconstructive and Oncoplastic surgery for giant Phyllodes tumors: a single center’s experience. World J Plast Surg.

[12] Rayzah M (2020). Phyllodes tumors of the breast: a literature review. Cureus.

[13] Zhao J, Gao M, Ren Y, Cao S, Wang H, Ge R (2021). A Giant borderline Phyllodes tumor of breast with skin ulceration leading to non-insular tumorigenic hypoglycemia: a case report and literature review. Front Endocrinol.

[14] Panda KM, Naik R (2016). A Clinicopathological study of benign phyllodes tumour of breast with emphasis on unusual features. J Clin Diagn Res.

[15] Spitaleri G, Toesca A, Botteri E (2013). Breast phyllodes tumor: a review of literature and a single center retrospective series analysis. Crit Rev Oncol Hematol.

[16] Tan PH, Ellis I, Allison K (2020). The 2019 World Health Organization classification of tumours of the breast. Histopathology.

[17] Ross DS, Giri DD, Akram MM (2017). Fibroepithelial lesions in the breast of adolescent females: a Clinicopathological study of 54 cases. Breast J.

[18] Zhang Y, Kleer CG (2016). Phyllodes tumor of the breast: histopathologic features, differential diagnosis, and molecular/genetic updates. Arch Pathol Lab Med.

[19] Koh VCY, Thike AA, Tan PH (2017). Distant metastases in phyllodes tumours of the breast: an overview. Applied Cancer Research.

[20] Wei J, Tan YT, Cai YC (2014). Predictive factors for the local recurrence and distant metastasis of phyllodes tumors of the breast: a retrospective analysis of 192 cases at a single center. Chin J Cancer.

[21] Zelek L, Llombart-Cussac A, Terrier P (2003). Prognostic factors in primary breast sarcomas: a series of patients with long-term follow-up. JCO.

[22] Rosenberger LH, Thomas SM, Nimbkar SN (2021). Contemporary Multi-Institutional Cohort of 550 Cases of Phyllodes Tumors (2007–2017) Demonstrates a Need for More Individualized Margin Guidelines. J Clin Oncol.

[23] Lee JS, Yoon K, Onyshchenko M (2021). Sarcoma of the breast: clinical characteristics and outcomes of 991 patients from the national cancer database. Sarcoma.

[24] Karczmarek-Borowska B, Bukala A, Syrek-Kaplita K, Ksiazek M, Filipowska J, Gradalska-Lampart M (2015). A Rare Case of Breast Malignant Phyllodes Tumor With Metastases to the Kidney: Case Report. Medicine.

[25] Perry M, Kelly N, Loughrey M, Hyland M, Caddy G (2012). Lumps, bumps and GI bleeding. Ulst Med J.

[26] Liu HP, Chang WY, Hsu CW (2020). A giant malignant phyllodes tumor of breast post mastectomy with metastasis to stomach manifesting as anemia: A case report and review of literature. BMC Surg.

[27] Choi DIl, Chi HS, Lee SH (2017). A Rare case of phyllodes tumor metastasis to the stomach presenting as anemia. Cancer Res Treat.

[28] Lim SZ, Selvarajan S, Thike AA (2016). Breast sarcomas and malignant phyllodes tumours: comparison of clinicopathological features, treatment strategies, prognostic factors and outcomes. Breast Cancer Res Treat.

[29] Robles-Tenorio A, Solis-Ledesma G (2022). Undifferentiated Pleomorphic Sarcoma.

[30] Gambichler T, Horny K, Mentzel T, et al. Undifferentiated pleomorphic sarcoma of the breast with neoplastic fever: case report and genomic characterization. J Cancer Res Clin Oncol. Published online May 2022. 10.1007/s00432-022-04000-6. 10.1007/s00432-022-04000-6 PMC10020307 35501497

[31] Rakha EA, Brogi E, Castellano I, Quinn C. Spindle cell lesions of the breast: a diagnostic approach. Virchows Archiv. Published online July 2021. 10.1007/s00428-021-03162-x. 10.1007/s00428-021-03162-x PMC8983634 34322734

[32] Lee JS, Yoon K, Onyshchenko M (2021). Sarcoma of the breast: clinical characteristics and outcomes of 991 patients from the national cancer database. Sarcoma.

[33] Tian H, Lian R, Li Y (2020). AKT-induced lncRNA VAL promotes EMT-independent metastasis through diminishing Trim16-dependent Vimentin degradation. Nat Commun.

[34] Co M, Chen C, Tsang JY, Tse G, Kwong A (2018). Mammary phyllodes tumour: a 15-year multicentre clinical review. J Clin Pathol.

[35] Spanheimer PM, Murray MP, Zabor EC (2019). Long-term outcomes after surgical treatment of malignant/borderline phyllodes tumors of the breast. Ann Surg Oncol.

[36] Noordman PCW, Klioueva NM, Weimann MN, Borgstein PJ, Vrouenraets BC (2020). Phyllodes tumors of the breast: a retrospective analysis of 57 cases. Breast Cancer Res Treat.

[37] Noronha Y, Raza A, Hutchins B (2011). CD34, CD117, and Ki-67 expression in phyllodes tumor of the breast: an immunohistochemical study of 33 cases. Int J Surg Pathol.

[38] Esposito NN, Mohan D, Brufsky A, Lin Y, Kapali M, Dabbs DJ (2006). Phyllodes tumor: A clinicopathologic and immunohistochemical study of 30 cases. Arch Pathol Lab Med.

[39] Tan PH, Jayabaskar T, Yip G (2005). p53 and c-kit (CD117) protein expression as prognostic indicators in breast phyllodes tumors: a tissue microarray study. Mod Pathol.

[40] Dunne B, Lee AHS, Pinder SE, Bell JA, Ellis IO (2003). An Immunohistochemical study of metaplastic spindle cell carcinoma, phyllodes tumor and fibromatosis of the breast. Hum Pathol.

[41] Sannino G, Marchetto A, Ranft A (2019). Gene expression and immunohistochemical analyses identify SOX2 as major risk factor for overall survival and relapse in Ewing sarcoma patients. EBioMedicine.

[42] Basu-Roy U, Seo E, Ramanathapuram L (2011). Sox2 maintains self renewal of tumor-initiating cells in osteosarcomas. Oncogene.

[43] Metz EP, Wuebben EL, Wilder PJ (2020). Tumor quiescence: elevating SOX2 in diverse tumor cell types downregulates a broad spectrum of the cell cycle machinery and inhibits tumor growth. BMC Cancer.

[44] Yonemori K, Hasegawa T, Shimizu C (2006). Correlation of p53 and MIB-1 expression with both the systemic recurrence and survival in cases of phyllodes tumors of the breast. Pathol Res Pract.

[45] Zhu G, Pan C, Bei JX (2020). Mutant p53 in cancer progression and targeted therapies. Front Oncol.

[46] Bogach J, Shakeel S, Wright FC, Hong NJL (2021). Phyllodes tumors: a scoping review of the literature. Anna Surg Oncol.

[47] Rodríguez-Núñez P, Romero-Pérez L, Amaral AT (2020). Hippo pathway effectors YAP1/TAZ induce an EWS–FLI1-opposing gene signature and associate with disease progression in Ewing sarcoma. J Pathol.

[48] Dokala A, Thakur SS (2017). Extracellular region of epidermal growth factor receptor: a potential target for anti-EGFR drug discovery. Oncogene.

[49] Tanaka K, Ozaki T (2018). New TNM classification (AJCC eighth edition) of bone and soft tissue sarcomas: JCOG Bone and Soft Tissue Tumor Study Group. Jpn J Clin Oncol.

[50] Adem C, Reynolds C, Ingle JN, Nascimento AG (2004). Primary breast sarcoma: clinicopathologic series from the Mayo Clinic and review of the literature. Br J Cancer.

[51] Thind A, Patel B, Thind K (2020). Surgical margins for borderline and malignant phyllodes tumours. Ann R Coll Surg Engl.

[52] Zhao W, Tian Q, Zhao A (2021). The role of adjuvant radiotherapy in patients with malignant phyllodes tumor of the breast: a propensity-score matching analysis. Breast cancer (Tokyo, Japan).

[53] Efared B, Ebang GA, Tahiri L (2018). Phyllodes tumors of the breast: Clinicopathological analysis of 106 cases from a single institution. Breast Dis.

[54] NCCN Guidelines® Insights: Breast cancer, phyllodes tumors of the breast.Version 4.2023. J Natl Compr Canc Netw. 2023;21(6):594–608. 10.6004/jnccn.2023.0031.

[55] Adesoye T, Neuman HB, Wilke LG, Schumacher JR, Steiman J, Greenberg CC (2016). Current trends in the management of Phyllodes tumors of the breast. Ann Surg Oncol.

[56] Barrow BJ, Janjan NA, Gutman H (1999). Role of radiotherapy in sarcoma of the breast–a retrospective review of the M.D. Anderson experience. Radiother Oncol.

[57] Mitus JW, Blecharz P, Jakubowicz J, Reinfuss M, Walasek T, Wysocki W (2019). Phyllodes tumors of the breast. The treatment results for 340 patients from a single cancer centre. Breast (Edinburgh, Scotland).

[58] Brodowicz T, Amann G, Leithner A (2012). Consensus diagnosis and therapy of soft tissue sarcoma. Wien Klin Wochenschr.

[59] Palta M, Morris CG, Grobmyer SR, Copeland ; Edward M., Mendenhall NP. Angiosarcoma After Breast-Conserving Therapy Long-Term Outcomes With Hyperfractionated Radiotherapy. 10.1002/cncr.24995. 10.1002/cncr.24995 20162708

[60] Park HJ, Ryu HS, Kim K, Shin KH, Han W, Noh DY (2019). Risk factors for recurrence of malignant phyllodes tumors of the breast. In Vivo.

[61] Kim YJ, Kim K (2017). Radiation therapy for malignant phyllodes tumor of the breast: An analysis of SEER data. Breast (Edinburgh, Scotland).

[62] Haussmann J, Corradini S, Nestle-Kraemling C (2020). Recent advances in radiotherapy of breast cancer. Radiat Oncol.

[63] Gutkin PM, Ganjoo KN, Lohman M (2020). Angiosarcoma of the breast: management and outcomes. Am J Clin Oncol.

[64] Kokkali S, Moreno JD, Klijanienko J, Theocharis S (2022). Clinical and molecular insights of radiation-induced breast sarcomas: is there hope on the horizon for effective treatment of this aggressive disease?. Int J Mol Sci.

[65] Linthorst M, van Geel AN, Baartman EA (2013). Effect of a combined surgery, re-irradiation and hyperthermia therapy on local control rate in radio-induced angiosarcoma of the chest wall. Strahlenther Onkol.

[66] Belkacémi Y, Bousquet G, Marsiglia H (2008). Phyllodes tumor of the breast. Int J Radiat Oncol Biol Phys.

[67] Bajpai J, Susan D (2016). Adjuvant chemotherapy in soft tissue sarcomas…Conflicts, consensus, and controversies. South Asian journal of cancer.

[68] Bousquet G, Confavreux C, Magné N (2007). Outcome and prognostic factors in breast sarcoma: a multicenter study from the rare cancer network. Radiother Oncol.

[69] Zer A, Prince RM, Amir E, Abdul Razak AR (2018). Multi-agent chemotherapy in advanced soft tissue sarcoma (STS) - A systematic review and meta-analysis. Cancer Treat Rev.

[70] Miyazaki C, Shiozawa M, Koike R (2019). Neoadjuvant chemotherapy for primary sarcoma of the breast: a case report. J Med Case Reports.

[71] Rosenbaum E, Antonescu CR, Smith S (2022). Clinical, genomic, and transcriptomic correlates of response to immune checkpoint blockade-based therapy in a cohort of patients with angiosarcoma treated at a single center. J Immunother Cancer.

[72] Cohen PR, Prieto VG, Kurzrock R (2021). Tumor lysis syndrome: introduction of a cutaneous variant and a new classification system. Cureus.

[73] Wang F, Jia Y, Tong Z (2015). Comparison of the clinical and prognostic features of primary breast sarcomas and malignant phyllodes tumor. Jpn J Clin Oncol.

[74] Gutkin PM, Ganjoo KN, Lohman M (2020). Angiosarcoma of the breast: management and outcomes. Am J Clin Oncology.

[75] Sherman KL, Kinnier CV, Farina DA (2014). Examination of national lymph node evaluation practices for adult extremity soft tissue sarcoma. J Surg Oncol.

[76] Ho BT, Shen J (2020). Periductal stroma sarcoma of the breast. Breast J.

[77] Khoury T, Gaudioso C, Fang YV (2018). The role of skin ulceration in breast carcinoma staging and outcome. Breast J.

[78] Fields RC, Aft RL, Gillanders WE, Eberlein TJ, Margenthaler JA (2008). Treatment and outcomes of patients with primary breast sarcoma. Am J Surg.

