# Supplementary material for: Clinical perspectives and outcomes of the giant breast phyllodes tumor and sarcoma: a real-world retrospective study
Source: BMC Cancer. 2023 Aug 28;23:801. doi: 10.1186/s12885-023-11279-2 (PMC10463853; doi:10.1186/s12885-023-11279-2)
Supplement: Supplementary file 1 — Additional file 1: Supplement 1. Hematoxylin and eosin staining of the giant sarcoma with negative result (original magnification, 200×) [file 12885_2023_11279_MOESM1_ESM.docx]

**Supplement 1**

**A B**


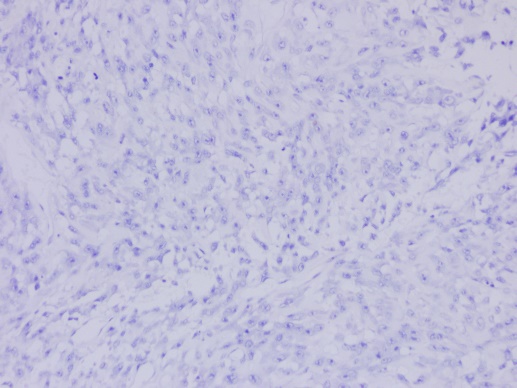

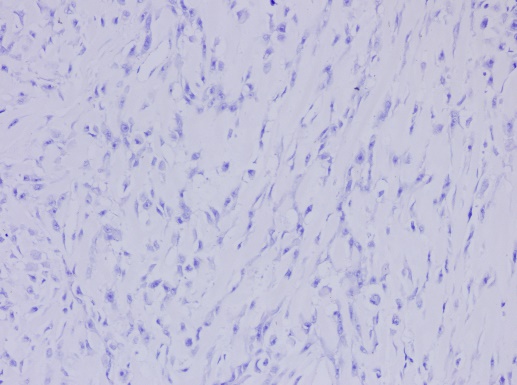


**C D**


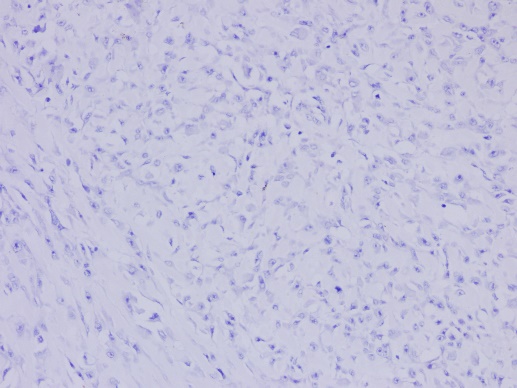

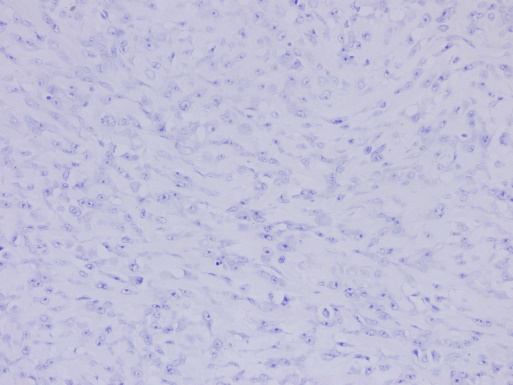


**E F**


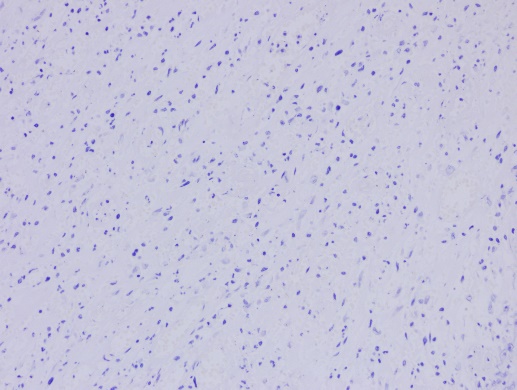

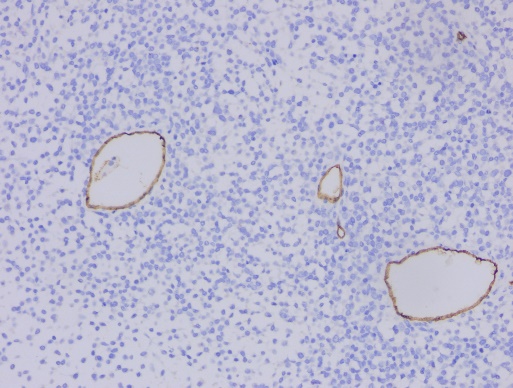


**G H**


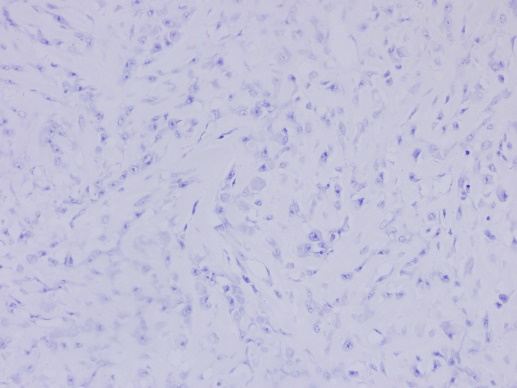

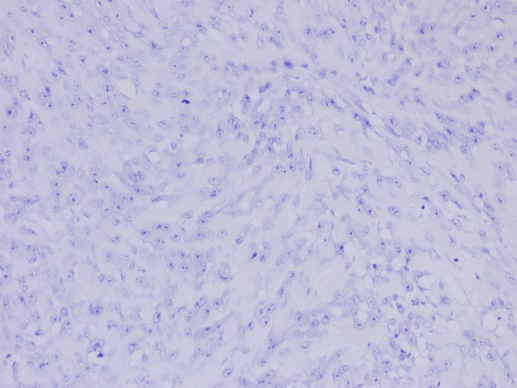


**I J**


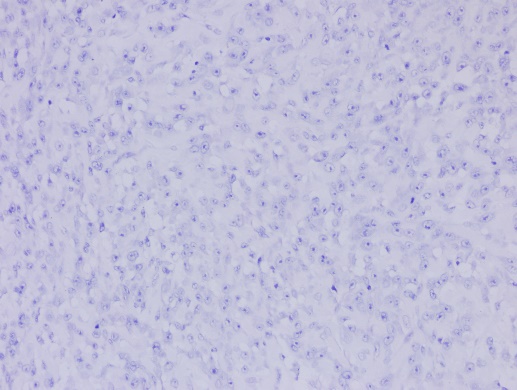

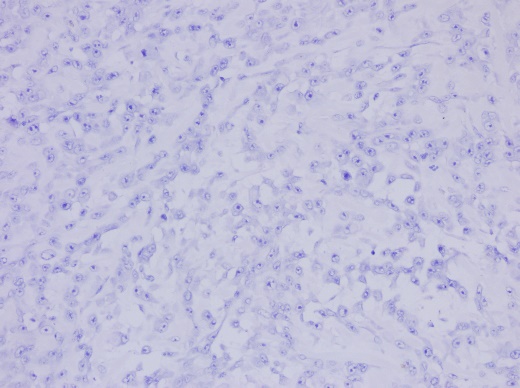


**K**

**
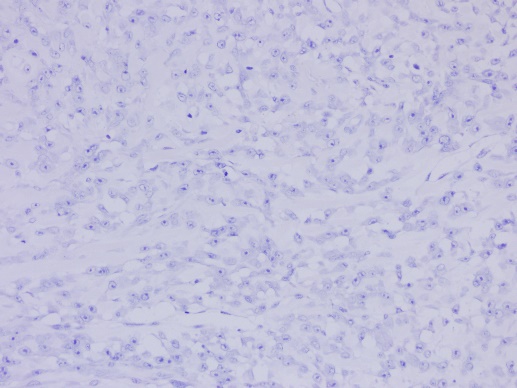
**

Supplement 1 Hematoxylin and eosin staining of the giant sarcoma with negative result (original magnification, 200×)

(A) CK

(B) Desmin

(C) EMA

(D) HMB-45

(E) Calponin

(F) CD34

(G) MelanA

(H) P63

(I) S-100

(J) Stat6

(K) β-catenin
